# EnsembleNPPred: A Robust Approach to Neuropeptide Prediction and Recognition Using Ensemble Machine Learning and Deep Learning Methods

**DOI:** 10.3390/life15071010

**Published:** 2025-06-25

**Authors:** Supatcha Lertampaiporn, Warin Wattanapornprom, Chinae Thammarongtham, Apiradee Hongsthong

**Affiliations:** 1 Biochemical Engineering and Systems Biology Research Group, National Center for Genetic Engineering and Biotechnology, National Science and Technology Development Agency at King Mongkut’s University of Technology Thonburi, Bangkok 10150, Thailand; supatcha.ler@biotec.or.th (S.L.); chinae19@gmail.com (C.T.); 2 Applied Computer Science Program, Department of Mathematics, Faculty of Science, King Mongkut’s University of Technology Thonburi, Bangkok 10150, Thailand; warin.wat@kmutt.ac.th

**Keywords:** neuropeptide prediction, prediction model, bioinformatics, ensemble learning, machine learning, combining methods, computational peptide discovery, deep learning, voting

## Abstract

Neuropeptides (NPs) are a diverse group of signaling molecules involved in regulating key physiological processes such as pain perception, stress response, mood, appetite, and circadian rhythms. Acting as neurotransmitters, neuromodulators, or neurohormones, they play a critical role in modulating and fine-tuning neural signaling networks. Despite their biological significance, identifying NPs through experimental techniques remains time-consuming and resource-intensive. To support this effort, computational prediction tools have emerged as a cost-effective approach for prioritizing candidate sequences for experimental validation. In this study, we propose EnsembleNPPred, an ensemble learning framework that integrates traditional machine learning (ML) models with a deep learning (DL) component. By combining the complementary strengths of these approaches, the model aims to improve generalization and predictive robustness. EnsembleNPPred employs a majority voting mechanism to aggregate the outputs from three classifiers: Support Vector Machine (SVM), Extra Trees (ET), and a CNN-based DL model. When evaluated on independent datasets, EnsembleNPPred demonstrated consistently competitive performance, achieving improvements in both accuracy and sensitivity-specificity balance compared to several existing methods. Furthermore, testing on multiple neuropeptide families from the NeuroPep database yielded an average accuracy of 91.92%, suggesting the model’s potential to generalize across diverse peptide classes. These results suggest that EnsembleNPPred may be a useful tool for early-stage neuropeptide candidate identification and for supporting downstream experimental validation.

## 1. Introduction

Neuropeptides are intercellular signaling molecules that function as neurotransmitters, neuromodulators, or neurohormones, playing crucial roles in regulating physiological processes such as pain perception, stress response, mood regulation, appetite control, and circadian rhythm maintenance [1,2]. By modulating the release of classical neurotransmitters and interacting with specific receptors—primarily G protein-coupled receptors (GPCRs)—they fine-tune neural activity across diverse systems [3,4,5]. Typically derived from larger precursor proteins (prepropeptides), neuropeptides undergo proteolytic processing and post-translational modifications within the regulated secretory pathway of neurons. These peptides are stored in dense-core vesicles and released in a stimulus-dependent manner. A single precursor can encode multiple copies of the same neuropeptide or distinct neuropeptides with different biological actions [4,5,6]. Notably, secreted peptides (e.g., hormones, growth factors) may share sequence similarity but are not considered classical neuropeptides unless they meet key criteria: neuronal origin, regulated release, and receptor-mediated neural signaling [2,4].

Neuropeptides have been identified in both vertebrates and invertebrates. Well-known examples include oxytocin, vasopressin, orexins, bradykinin, neuropeptide Y, tachykinin, opioids, somatostatin, angiotensin, calcitonin, gastrin, and galanin, which regulate functions such as growth, glucose homeostasis, inflammation, stress, reproduction, and memory [7,8]. Dysregulation of neuropeptides has been implicated in several diseases, including Alzheimer’s disease, Parkinson’s disease, epilepsy, diabetes, hypertension, and dermatological disorders [9]. Recent studies have also identified dual-function neuropeptides that exhibit both canonical signaling roles and antimicrobial activity, often linked to immune modulation [10,11]. These peptides participate in host immunity through bidirectional communication between the brain and gut, frequently involving microbiome interactions under biotic- and/or abiotic stress conditions [12]. In addition to their conserved functional domains, many of these peptides contain N-terminal signal sequences or domains that facilitate secretion and cellular targeting [13,14].

With the advancement of high-throughput sequencing technologies, the number of sequenced genomes has expanded rapidly, offering new opportunities to identify putative neuropeptides across diverse species. Although mass spectrometry remains the gold standard for neuropeptide characterization, it is labor-intensive and resource demanding. In this context, computational prediction methods offer an efficient and cost-effective complement, facilitating high-throughput candidate screening and accelerating the pace of neuropeptide discovery.

The availability of curated resources such as NeuroPep [15], which has expanded from 5949 peptides in 2015 to over 11,000 entries in its latest release [16], has facilitated the development of machine learning (ML) and deep learning (DL) models for neuropeptide prediction. Previous methods, including NeuroPIpred [17], PredNeuroP [18], NeuroPpred-Fuse [19], NeuroPred-FRL [20], NeuroPred-CLQ [21], and NeuroPred-PLM [22], have introduced innovative strategies in feature engineering and learning frameworks, contributing significantly to computational neuropeptide discovery. These models have laid a strong foundation, though challenges remain, particularly in improving generalizability and minimizing false positives across diverse peptide families.

Recent studies highlight the growing integration of deep learning (DL), emphasizing its ability to capture complex sequential dependencies and hierarchical representations that traditional ML may overlook. DL models such as convolutional neural networks (CNNs), recurrent neural networks (RNNs), long short-term memory (LSTM), and bidirectional LSTM (BiLSTM) have been increasingly utilized with minimal manual feature engineering [23,24,25,26,27]. Additionally, hybrid approaches that combine DL with traditional ML techniques have emerged, leveraging both the abstraction capability of deep models and the interpretability of classical algorithms [23,26,27].

In this study, we propose EnsembleNPPred, a hybrid neuropeptide prediction framework that integrates interpretable handcrafted ML features with CNN-derived representations from Word2Vec-encoded peptide sequences. By combining the complementary strengths of handcrafted features and automatically learned deep embeddings, the ensemble model aims to improve both predictive accuracy and interpretability. The ensemble strategy also enhances generalization, improves robustness to feature variability, and supports flexible deployment across different computational environments by selectively utilizing either ML or DL components. The integration may also aid in prioritizing candidates for downstream experimental validation.

Evaluation on two independent test sets demonstrates that EnsembleNPPred consistently achieves strong predictive performance in terms of accuracy, Matthews correlation coefficient (MCC), and AUC, while maintaining competitive sensitivity and specificity. Additionally, performance on a third dataset encompassing a wide range of neuropeptide families yielded an overall accuracy of 91.92%, underscoring its potential for broad applicability in neuropeptide prediction.

## 2. Materials and Methods

**Figure 1:**
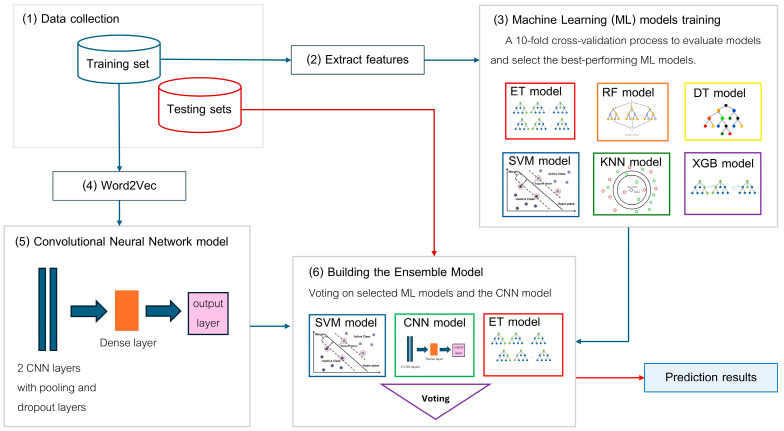
Workflow of EnsembleNPPred. Blue arrows indicate training flow; red arrows indicate testing or evaluation flow. Different colored borders represent individual ML models.

The key steps in the proposed EnsembleNPPred workflow are illustrated in Figure 1 and summarized as follows. (1) Data collecting involves assembling both training and testing datasets. (2) Feature extraction and selection are then performed to generate representative features suitable for machine learning. (3) A range of machine learning (ML) models, including Extra Trees (ET), Random Forest (RF), Decision Trees (DT), Support Vector Machine (SVM), k-Nearest Neighbors (KNN), and XGBoost (XGB), are trained and evaluated using 10-fold cross-validation to identify the top-performing models. (4) In parallel, a Word2Vec embedding approach is applied to encode peptide sequences for deep learning. (5) A convolutional neural network (CNN) model is constructed, trained, and fine-tuned for optimal performance. (6) Finally, an ensemble model is developed by combining the predictions from the selected ML models and the CNN model using a voting mechanism. The final prediction output is generated by voting strategy. This integrative pipeline leverages both traditional ML and DL techniques to enhance predictive performance for neuropeptide classification. The complete workflow is illustrated in Figure 1.

### 2.1. Dataset Preparation

Collecting a high-quality dataset is a crucial initial step in developing a reliable machine learning model. In this study, we utilized datasets from two sources: NeuroPred-CLQ [21] and NeuroPred-PLM [22]. To construct our training data, we adopted a strategy to minimize redundancy across datasets. As illustrated in Step 1 of the EnsembleNPPred workflow (Figure 1), we defined the training datasets from NeuroPred-CLQ and NeuroPred-PLM as *D*_1_ and *D*_2_, respectively, and their corresponding testing sets as *T*_1_ and *T*_2_. Since *D*_1_ may contain sequences also present in *T*_2_, and *D*_2_ may include sequences from *T*_1_, we defined the full dataset as the union:

*D* = *D*_1_ ∪ *D*_2_

*T* = *T*_1_ ∪ *T*_2_

The final training dataset was obtained by removing all sequences in the testing sets from the union of the training datasets, formally represented as:

CombinedTrainingSet = *D*\*T = (D*_1_ ∪ *D*_2_*)*\*(T*_1_ ∪ *T*_2_*)*

where “\” denotes set subtraction, ensuring that overlapping sequences between training and testing sets were excluded to prevent data leakage.

To further reduce redundancy, we applied CD-HIT with a 90% sequence identity threshold. This clustered highly similar sequences and retained only representatives with less than or equal to 90% identity, resulting in a non-redundant training dataset denoted as:

FilteredTrainingSet = CD-HIT_90%_(CombinedTrainingSet)

For model evaluation, we employed two independent test sets. Testing dataset 1, sourced from NeuroPred-CLQ [21], comprises 485 NPs and 485 non-NPs, and has been widely adopted in prior studies, enabling direct performance comparisons. Testing dataset 2, derived from NeuroPred-PLM [22], includes 444 unique NPs and 444 unique non-NPs.

To ensure independence of training and testing datasets and mitigate the risk of data leakage, we conducted BLASTP searches using the training set as the database and each test set as the query, applying a stringent E-value cutoff of 0.00001. For the NeuroPred-PLM test set, only 17.1% of queries returned significant hits (identity scores: 41.2–89.7%), while 82.9% showed no detectable similarity. Similarly, for the NeuroPred-CLQ test set, 27.8% had matches (identity scores: 36.8–89.9%), and 72.2% returned no significant hits. Importantly, no test sequences exhibited over 90% identity with any training sequence, validating the non-redundancy and independence of the datasets.

A detailed analysis of sequence length and physicochemical properties of the training dataset is presented in Section 3.1 (Amino acid composition and positional residue analysis). All datasets used in this work are publicly available at: http://www.ncrna-pred.com/EnsembleNPPred.htm (accessed on 24 May 2025) (http://www.ncrna-pred.com/Data.tar.xz (accessed on 24 May 2025)).

### 2.2. Feature Extraction and Feature Engineering

Various numerical representation schemes were employed to characterize peptide sequences, resulting in a feature vector comprising 982 numerical descriptors. These features are categorized into eight main types as follows:

(1) AAC descriptors: These represent the relative abundance of each amino acid type in a protein sequence. The proportions of all 20 standard amino acids [A, C, D, E, F, G, H, I, K, L, M, N, P, Q, R, S, T, V, W, Y] are calculated (AAC1-AAC20).
(2) Chou’s pseudo amino acid composition (PseAAC): PseAAC converts protein sequences of varying lengths into fixed-length numerical feature vectors, incorporating sequence-order information. Unlike AAC, PseAAC captures more detailed information, making it suitable for various sequence-based prediction tasks [28,29]. In this study, PseAAC was computed with parameters λ = 3 and weight = 0.05, resulting in 23 dimensions (PAAC1-PAAC23). Additional PseAAC variants were also calculated: parallel correlations (PsePC1-PsePC22), series correlations (PseSC1-PseSC26), and amphiphilic pseudo AACs based on hydrophobicity (APAAC1-APAAC23) and hydrophilicity correlation functions (APAAC24-APAAC46).
(3) CTD descriptors: These are derived from grouped amino acid compositions [30,31]. These include composition descriptors (CTDC1-CTDC21), transition descriptors (CTDT1-CTDT21), and distribution descriptors (CTDD1-CTDD105). These descriptors were calculated using the protr R package (1.7.4) [32], with amino acids classified into three groups based on seven physicochemical properties: normalized van der Waals volume, charge, hydrophobicity, polarity, secondary structure, and solvent accessibility.
(4) Quasi-sequence-order descriptors: These are based on the distance matrix of the 20 amino acids [33] and include sequence-order-coupling numbers (SOCN1-SOCN6) and quasi-sequence-order descriptors (QSO1-QSO46), computed with lag = 3 and weight = 0.1.
(5) Physicochemical and topological property-related features: These encompass the Crucian properties covariance index (Crucian1–Crucian3) [34], Z-scales (zscales1–zscales5) [35], factor analysis scales of generalized amino acid information (fasgai1–fasgai6) [36], T-scales (tScales1–tScales5) [37], VHSE-scales (vhsescales1–vhsescales8) [38], protFPs (protFP1–protFP8) [39], ST-scales (stscales1–stscales8) [40], MS-WHIM scores (mswhimscore1–mswhimscore3) [41], the aliphatic index (aIndex) [42], Geary autocorrelations (geary1–geary12), autocovariance index (autocov) [42], potential protein interaction index (Boman) [43], cross-covariance indices (Crosscov1–Crosscov2), net charge (Charge), instability index (Instaindex) [44], hydrophobic moment for alpha helices (Hmoment1), hydrophobic moment for beta sheets (Hmoment2), BLOSUM matrix-derived descriptors (Blosum1–8), and isoelectric point (pI) calculated using the peptide R package [45].
(6) Occurrence of 2-mer and selected 3-mer motifs: Initially, all possible 2-mers (400 dimensions) were generated and retained. Then, 3-mers (8000 dimensions) were generated, and only those significantly different between positive and negative data, as determined by log-odds and MERCI [46] scores, were selected. The selected 3-mer motifs include: ALP, DFI, DTD, ENL, ETI, FLP, FYP, GLQ, GPF, HLP, HPF, IAW, IFP, IKW, IPA, IPP, IYP, KDQ, KRI, KVL, LAV, LHL, LLE, LMR, MFL, NPC, NVP, NWN, PAG, PEV, PFP, PGA, PIP, PIT, PKH, PLP, PSE, PTH, PVP, PYP, QTP, RLN, RND, STC, TKE, TLE, TLV, TST, VKE, VLP, VPP, VPQ, VRP, VYP, WLP, YNP, and YST motifs.
(7) Secondary structure conformation features: The propensities for aggregation, amyloid formation, turns, alpha-helices, helical aggregation, and beta-strand structures were calculated using the Tango program [47] (tango1-tango6).
(8) Composite features for neuropeptides: To enhance prediction with more informative features, we implemented a method for generating composite features by combining significant attributes using a logistic regression model. Multiple composite features were developed and evaluated through a 10-fold cross-validation process (referred to as logistic1-logistic15). The detailed process for constructing these composite features is outlined in the hybrid feature section of ensemble-AMPPred [48]. A set of selected features was used to fit a logistic regression model, which is expressed by the following equation:

$$\mathrm{Prob}.\;(Y=\mathrm{NPs}|x)=\mathrm{logistic}\;(x)=\frac{e^{\beta_{0}+\beta_{1}X_{1}+\beta_{2}X_{2}+\beta_{3}X_{3}+\cdots +\beta nXn}\;}{1+e^{\beta_{0}+\beta_{1}\;X_{1}+\beta_{2}X_{2}+\beta_{3}X_{3}+\cdots +\beta nXn}}$$

A logit transformation (natural logarithm of the odds ratio for Y being in the NPs category) was applied to establish a link function with the logistic regression model. The logit function is defined as follows:

$$\mathrm{Logit}(x)=\log(\frac{P\left(Y=NPs\;\right|X=X)}{P\left(Y=nonNPs|x=x\right)})=\beta_{0}+\beta_{1}X_{1}+\beta_{2}X_{2}+\beta_{3}X_{3}+\cdots +\beta_{n}X_{n}$$

As a result, the composite feature is represented by the following equation:

Composite Feature = *β*_0_ + *β*_1_ *feature1* + *β*_2_*feature2* + *β*_3_*feature3* + ⋯ + *β_n_ featureN*

In this equation, β_0_ denotes the intercept, while β_1_, β_2_, β_3_, and β_n_ correspond to the regression coefficients of each selected feature. The variables feature1, feature2, …, and featureN represent the individual features that make up the composite feature. Among the selected composite features, logistic1 demonstrated the highest sensitivity and is expressed as follows:

$$\mathrm{logistic}1=\beta_{0}+\beta_{1}mer5+\beta_{2}kmer195+\beta_{3}Kmer285+\beta_{4}Kmer92+\beta_{5}Pse\_SC16+\beta_{6}Charge$$

where β_0_ is the intercept, β_1_, β_2_, β_3_, β_4_, β_5_, and β_6_ represent the regression coefficients; mer5 is the amino acid composition of Phe (F) in a peptide; kmer195 is the di-amino-acid composition of LR; kmer285 is the di-amino-acid composition of RF; and kmer92 is the di-amino-acid composition of FN. Pse_SC16 is a pseudo amino acid composition (PseAAC) descriptor in the generalized mode of amino acid Ser (S). Charge is a computed net charge of a peptide.

### 2.3. Feature Selection

To identify a discriminative subset of features, a feature selection (FS) method was employed to select relevant and informative features that efficiently discriminate NPs from non-NPs. The ReliefF [49] method was applied as a preprocessing step to eliminate irrelevant features. This method assigns a ReliefF score to each feature based on its ability to differentiate between neighboring instances from different classes. Features that contribute more significantly to separating samples were assigned higher weights, prioritizing those that enhance local class discrimination. To assess potential multicollinearity among features, we computed Pearson correlation coefficients for all possible feature pairs. Among 481,671 possible combinations, only 621 pairs (0.13%) exhibited high correlation (|r| ≥ 0.8), indicating minimal redundancy. This supports the suitability of the ReliefF method, which prioritizes feature relevance over correlation.

### 2.4. Base Classifier Selection and Model Implementation

To construct a robust ensemble classifier, we evaluated six machine learning (ML) algorithms: Support Vector Machine (SVM) [50], k-Nearest Neighbors (kNN) [51], Decision Tree (DT) [52], Random Forest (RF) [53], Extreme Gradient Boost (XGB) [54], and Extra Trees (ET) [55]. Each algorithm offers unique inductive biases and learning mechanisms, which, when combined in an ensemble, can contribute to improved prediction diversity and generalization. Hyperparameter tuning was conducted using grid search to determine the optimal configuration for each model.

The SVM model is a supervised learning algorithm used for both classification and regression tasks. It maps data into a high-dimensional space and identifies a hyperplane that maximizes the margin between classes. In this study, we used a radial basis function (RBF) kernel with the following parameters: C = 19 and gamma = 0.125.

The kNN classifier is a non-parametric method used in statistical pattern recognition. It classifies a data point based on the majority label among its k-Nearest Neighbors. We used k = 9 and applied inverse distance weighting to compute similarity.

The Decision Tree (DT) is a tree-based classifier that uses a set of hierarchical decision rules to partition the feature space. It is valued for its interpretability and low computational cost. The confidence factor was set to 0.25 in our implementation.

The Random Forest (RF) algorithm is an ensemble of decision trees that aggregates predictions via majority voting. It is known for high accuracy and robustness to overfitting, especially on datasets with mixed feature types. We used 300 trees in our model.

The Extra Trees (ET) algorithm, or Extremely Randomized Trees, is another ensemble technique that introduces additional randomness during training. Unlike RF, which selects optimal split from a random subset of features, ET randomly selects both features and split points, resulting in greater model diversity. This stochastic approach reduces variance and training time, making ET especially suitable for large or noisy datasets. In this study, we used 900 trees.

The Extreme Gradient Boosting (XGB) model is based on the gradient boosting framework. It iteratively trains weak learners, optimizing for a differentiable loss function by adjusting weights at each iteration. The following parameters were used: nrounds = 150, max_depth = 5, learning rate (eta) = 0.03, and subsample = 0.6.

We also implemented a deep learning model using a Convolutional neural network (CNN) [56,57], which is effective for identifying spatial patterns in grid-like biological data. Input peptide sequences were encoded using Word2Vec embeddings [58] with the skip-gram approach to represent k-mers in a 120-dimensional vector space.

We empirically explored several CNN architectures with 2 to 6 convolutional layers and also evaluated LSTM-based models. Although deeper networks had higher capacity, they showed diminishing returns in performance and increased overfitting risk on the training data. The final CNN configuration was selected based on empirical results and tuned via grid search with five-fold cross-validation. The selected architecture consisted of two convolutional layers (64 and 32 filters; kernel size = 3), each followed by a max pooling layer (pool size = 2), and ReLU activation functions, followed by a fully connected dense layer (32 units, ReLU activation) and a dropout rate of 0.3. The model was optimized using Adam’s optimizer with a learning rate of 0.0001 for up to 100 epochs with early stopping applied. This CNN module automatically learns hierarchical, discriminative sequence features analogous to autoencoding, effectively reducing the need for manual feature engineering. It complements traditional handcrafted features with learned nonlinear representations to improve classification.

All implementations were performed using a combination of Perl (v5.38.2), Python (v3.12.6), Weka (v3.8.6; University of Waikato, Hamilton, New Zealand), and R (v4.2.2; R Foundation for Statistical Computing, Vienna, Austria) on a Fedora Linux-based system (Fedora 39; Red Hat, Inc., Raleigh, NC, USA) equipped with an Intel Core i7 CPU @ 2.30 GHz, 16 GB RAM, and an NVIDIA GeForce RTX3070 GPU. The trained models and standalone program are publicly available at: http://www.ncrna-pred.com/EnsembleNPPred.htm (accessed on 24 June 2025).

A 10-fold cross-validation was conducted on the training dataset to evaluate classification performance and identify the best-performing models for integration into the final program. Subsequently, two independent test datasets were used to validate the selected models and benchmark performance against existing neuropeptide prediction tools.

The model’s performance was assessed using the following evaluation metrics:

$$Accuracy(ACC)=\frac{TP+TN}{\left(TP+TN+FP+FN\right)}$$

$$Sensitivity(Recall,Sn)=\frac{TP}{\left(TP+FN\right)}$$

$$Specificity(Sp)=\frac{TN}{\left(TN+FP\right)}$$

$$Precision=\frac{TP}{\left(TP+FP\right)}$$

$$MatthewsCorrelationCoefficient(MCC)=\frac{TP\times TN-FP\times FN}{\sqrt{\left(TP+FP)\times (TP+FN)\times (TN+FP)\times (TN+FN\right)}}$$

$$F1score=2\times \frac{Precision\times Recall}{Precision+Recall}$$

where TP, TN, FP, and FN represent true positives, true negatives, false positives, and false negatives, respectively. These metrics were used to evaluate the model’s overall accuracy, class balance, and predictive reliability.

In addition, a Receiver Operating Characteristic (ROC) curve was generated to illustrate the trade-off between sensitivity and specificity across various classification thresholds. The Area Under the Curve (AUC) was calculated to assess the classifier’s ability to distinguish between classes, with an AUC of 1.0 indicating a perfect classifier.

## 3. Results and Discussion

### 3.1. Amino Acid Composition and Positional Residue Analysis

The amino acid composition of neuropeptides (NPs) reveals distinguishing distinctive properties that differentiate them from non-neuropeptides (non-NPs). Table 1 summarizes the percentage of amino acid groups, categorized by physicochemical properties, as calculated using COPid [59]. Neuropeptides tend to be shorter in length and are enriched in aromatic, polar, and negatively charged residues, which may be crucial for bioactivity, receptor interaction, and molecular recognition. These compositional features provide valuable input for machine learning models aimed at distinguishing neuropeptides from other peptide sequences.

**Figure 2:**
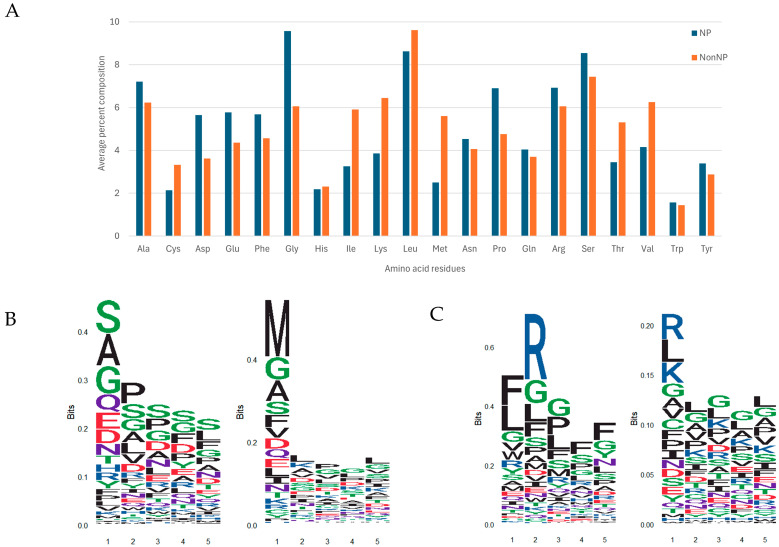
Composite analysis of amino acid composition and conserved sequence motifs in neuropeptides (NPs) vs. non-neuropeptides (non-NPs). (**A**) Average percent composition of amino acid residues in NP and non-NP datasets. (**B**) Sequence logos plots showing amino acid enrichment at positions 1–5 of the N-terminal region in the training data. Left panel: NP-positive dataset. Right panel: NP-negative dataset. (**C**) Sequence logos plots showing amino acid enrichment at positions 1–5 of the C-terminal region in the training data. Left panel: NP-positive dataset. Right panel: NP-negative dataset. Color codes for amino acids in (**B**,**C**): Red: acidic residues (D, E); Blue: basic residues (K, R, H); Purple: neutral residues (Q, N); Black: hydrophobic residues (A, V, L, I, P, W, F, M); Green: polar residues (G, S, T, Y, C). The height of each letter represents the relative frequency (information content) of the amino acid at the designated position.

Figure 2A presents a comparison of the average amino acid composition between neuropeptides (NPs) and non-neuropeptides (non-NPs). The analysis reveals notable differences in the relative abundance of specific residues. Amino acids such as Glycine (G), Proline (P), Phenylalanine (F), Serine (S), and Arginine (R) are more abundant in NPs, suggesting their potential involvement in neuropeptide functionality, possibly contributing to flexibility, receptor interaction, or bioactivity. Conversely, residues including Valine (V), Threonine (T), Methionine (M), Cysteine (C), Lysine (K), Isoleucine (I), and Leucine (L) are more prevalent in non-NPs, which may reflect their association with structural stability or non-signaling peptide segments. These compositional differences emphasize underlying physicochemical properties that may influence neuropeptide behavior and can be informative for classification tasks.

Positional residue analysis of both the N-terminal and C-terminal regions was conducted by calculating the average amino acid composition at positions 1 to 5 for each terminus in neuropeptides (NPs, positive) and non-neuropeptides (non-NPs, negative). As illustrated in Figure 2B,C, sequence logos were generated using ggseqlogo [60]. In these logos, amino acids are color-coded as follows: red for acidic, blue for basic, purple for neutral, black for hydrophobic, and green for polar residues. The height of each letter reflects the relative frequency of that amino acid at the corresponding position.

Figure 2B shows the first five N-terminal residues for both NPs (left panel) and non-NPs (right panel). The NP panel shows a notable preference for polar (green) and acidic (red) residues, suggesting sequence conservation at the N-terminus. In contrast, the non-NP panel displays a more uniform distribution without a clear dominant residue pattern, suggesting less positional conservation.

Figure 2C displays the amino acid distribution at the C-terminal region. The NP panel shows a clear enrichment of hydrophobic (black), polar (green), and basic (blue) residues, particularly arginine (R) and glycine (G), along with phenylalanine (F), leucine (L), proline (P), and serine (S). By contrast, the non-NP panel shows a broader, less structured distribution, further suggesting a lack of motif conservation at the C-terminus in non-neuropeptides.

The most prevalent amino acids at the N-terminus of NPs were Serine (S, 11.70%), Glycine (G, 9.32%), Alanine (A, 8.40%), Proline (P, 8.37%), Asparagine (N, 5.18%), and Tyrosine (Y, 4.07%). At the C-terminus, the dominant residues were Phenylalanine (F, 13.80%), Leucine (L, 10.45%), Arginine (R, 10.29%), and Glycine (G, 9.95%).

**Figure 3:**
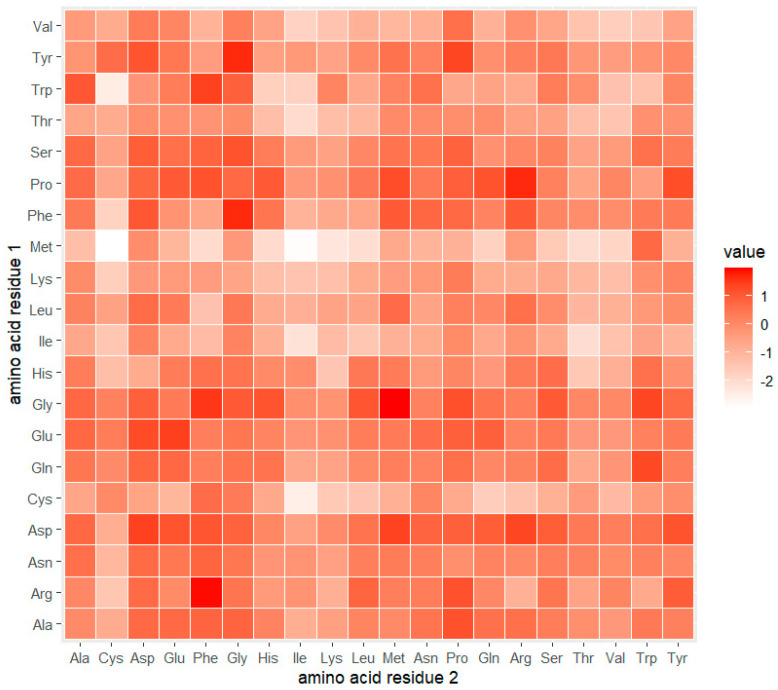
Heatmap of log-odds ratios for dipeptide (2-mer) frequencies comparing NP-positive and NP-negative peptide sequences. Each cell represents a dipeptide, with the amino acid at position 1 on the *y*-axis and the amino acid at position 2 on the *x*-axis. Color legend: red shades indicate 2-mers that are overrepresented in NPs relative to non-NPs (positive log-odds values), while lighter red to white shades indicate underrepresentation in NPs (negative log-odds values). The color scale ranges approximately from −3 (underrepresented in NPs) to +3 (strongly enriched in NPs).

In addition, Figure 3 presents a heatmap of log-odds scores for 2-mer (dipeptide) motifs comparing NPs vs. non-NPs. In this heatmap, each cell represents a specific amino acid pair, with the first residue on the y-axis and the second residue on the x-axis. The color intensity indicates the log-odds ratio; red shading indicates dipeptides overrepresented in NPs, while lighter red to white shading indicates lower or underrepresented occurrences in NPs relative to non-NPs. The log-odds values range approximately from −3 to +3, with zero indicating no difference between the two groups.

Notably, several dipeptides such as Gly-Phe (GF; 1.51), Phe-Gly (FG; 1.67), Arg-Phe (RF; 1.88), Tyr-Gly (YG; 1.67), Gly-Met (GM; 1.95), and Pro-Arg (PR; 1.67) were found to be overrepresented in NPs. These motifs suggest potential sequence features contributing to neuropeptide specificity. Conversely, dipeptides such as Met-Cys (MC; −2.98), Trp-Cys (WC; −2.49), Met-Phe (MF; −2.02), Met-Ile (MI; −2.91), Met-Lys (MK; −2.29), Met-Leu (ML; −2.08), Ile-Ile (II = −2.20), and Cys-Ile (CI; −2.53) were more prevalent in non-NPs, possibly indicating these pairings may be more characteristic of non-functional or structural peptides. Taken together, these findings highlight amino acid and dipeptide-level features that distinguish NPs from non-NPs, offering useful insights for improving predictive modeling and sequence-based classification of neuropeptides.

### 3.2. 10-Fold Cross-Validation of Predictive Performance with the Training Dataset

To evaluate the generalization performance of individual classifiers, we conducted 10-fold cross-validation on the training dataset. The key evaluation metrics including accuracy (ACC), Matthews correlation coefficient (MCC), sensitivity (Sn), specificity (Sp), area under the ROC curve (AUC), and 95% confidence intervals for the ROC (CI) are summarized in Table 2.

Among the individual models, the DL classifier achieved the highest accuracy (93.918%) and MCC (0.878), demonstrating strong overall performance. The ET and SVM models also performed competitively, with accuracy exceeding 93.7% and AUC values of 0.986. The RF and XGB classifiers followed closely, while the DT and KNN models showed relatively lower performance.

Based on these results, we constructed a heterogeneous ensemble model combining SVM, DL, and ET. This integration leveraged the complementary strengths of each classifier: DL contributed high overall metrics, SVM provided the highest sensitivity, and ET enhanced predictive diversity through its randomized feature subspaces.

The final ensemble model, which integrated predictions through a voting mechanism, achieved an accuracy of 93.978%, an MCC of 0.880, and an AUC of 0.987. It outperformed all individual models in terms of MCC and specificity (0.941), suggesting more balanced and robust predictions, particularly in reducing false positives, which is critical for distinguishing neuropeptides from non-neuropeptides. The ensemble’s narrow AUC confidence interval further supports its performance consistency across validation folds. These results align with the intended mitigation strategy embedded in our model design by combining diverse learners (SVM, ET, CNN) to reduce individual model bias and variance while enhancing predictive reliability. Additionally, the hybrid ensemble integrates interpretable handcrafted features with deep learning-derived sequence embeddings. While handcrafted features offer domain relevance and model transparency, the CNN applied to Word2Vec embeddings captures complex, nonlinear sequence patterns analogous to autoencoding. This complementary combination balances interpretability with feature richness, contributing to a performance-oriented and interpretable framework for neuropeptide classification.

**Figure 4:**
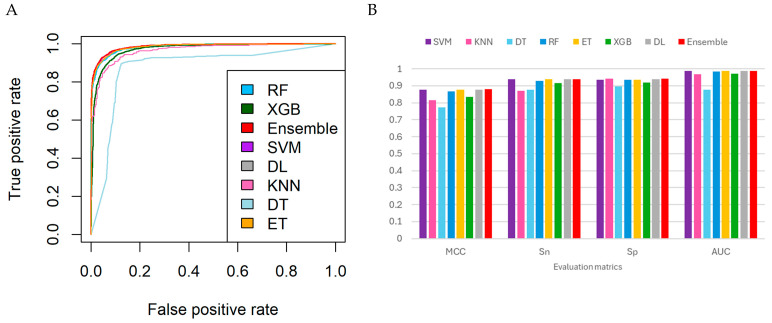
(**A**) Receiver operating characteristic (ROC) curves for individual classifiers and the ensemble models, evaluated using 10-fold cross-validation. The ensemble model shows the highest overall performance, followed closely by the Deep Learning (DL), Support Vector Machine (SVM), Extra Trees (ET), and Random Forest (RF) models. In contrast, the Decision Tree (DT) and k-Nearest Neighbors (KNN) models demonstrate lower AUC and sensitivity, indicating reduced classification reliability. (**B**) Bar chart comparing four key evaluation metrics: Matthews correlation coefficient (MCC), sensitivity (Sn, or true positive rate), specificity (Sp, or true negative rate), and area under the ROC curve (AUC) across all models. The *x*-axis represents the evaluation metrics, while the *y*-axis indicates the metric value ranging from 0 to 1. Colored bars correspond to each classifier as indicated in the legend. The ensemble model consistently achieves strong and balanced performance across all metrics, with notable improvements in MCC and specificity. SVM, DL, and ET models also perform competitively, while DT and KNN show weaker overall performance, especially in MCC.

Taken together, these findings suggest that the ensemble approach effectively combines high-performing base classifiers and demonstrates enhanced generalization capability. A comparison of ROC curves and cross-validation performance metrics is presented in Figure 4.

### 3.3. Feature Interpretability and Importance

**Figure 5:**
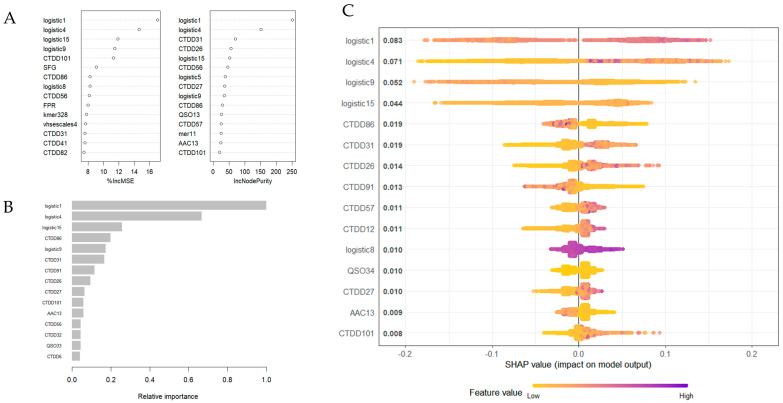
Feature importance analysis. (**A**) Feature importance rankings from the Random Forest model, measured by percent increase in mean squared error (%IncMSE) and node purity (IncNodePurity). (**B**) Relative importance scores of top-ranked features from the XGBoost model. (**C**) SHAP summary plot of the top 15 features. The *y*-axis lists features sorted by importance (highest to lowest). The *x*-axis indicates SHAP values, which reflect the change in log-odds used to derive predicted probabilities. Colors represent feature values. Logistic1 consistently ranked highest across all methods, followed by other composite logistic features and CTD-derived descriptors, supporting their critical role in distinguishing neuropeptides from non-neuropeptides.

Interpreting model behavior and identifying influential features are essential for validating predictions and gaining biological insights. Understanding which features contribute most to the model’s predictions can provide biological insights and help validate model behavior. Feature importance was initially assessed using the built-in feature ranking mechanisms of the RF and XGB models. In both models, the composite feature logistic1 consistently emerged as the most informative, as shown in Figure 5A,B.

To further investigate feature contributions, we conducted Shapley Additive exPlanations (SHAP) analysis. SHAP is a widely used game-theoretic approach that quantifies the impact of each feature on individual predictions [61]. The SHAP summary plot in Figure 5C supports the RF and XGB results, again highlighting logistic1 as the most influential feature.

The SHAP framework estimates the contribution of each input feature to the final prediction for each individual sample. By assigning importance values based on the mean absolute SHAP value across all predictions, SHAP provides a more detailed and individualized interpretation than traditional feature importance methods. These consistent findings across both tree-based methods (RF and XGB) and SHAP analysis indicate that composite features such as logistic1, along with CTD-derived descriptors, play a critical role in distinguishing neuropeptides from non-neuropeptides in the EnsembleNPPred model.

### 3.4. Performance Comparison of Various Existing Predictive Models

To assess the performance of the proposed EnsembleNPPred method, two independent testing datasets were used. The performance of our model was evaluated against several previously published neuropeptide prediction methods, using testing dataset 1 and testing dataset 2, as summarized in Table 3 and Table 4, respectively.

As shown in Table 3, the performance of various neuropeptide prediction models was assessed using five key metrics. NeuroPred-FRL achieved an accuracy (ACC) of 0.900, MCC of 0.803, sensitivity (Sn) of 0.946, specificity (Sp) of 0.854, and an AUC of 0.965. NeuroPpred-Fuse demonstrated good specificity (0.930) but had slightly lower sensitivity compared to NeuroPred-CLQ. NeuroPIpred showed the lowest performance across all metrics, with an ACC of 0.536, MCC of 0.074, Sn of 0.331, Sp of 0.736, and an AUC of 0.581. These results highlight the limited effectiveness of NeuroPIpred, which was specifically trained and designed for insect neuropeptides, when applied to broader datasets encompassing neuropeptides from various animal phyla. NeuroPred-CLQ exhibited strong performance with an ACC of 0.936, MCC of 0.875, Sn of 0.897, Sp of 0.975, and an AUC of 0.988. This model’s high specificity and AUC indicate its ability to correctly identify neuropeptides, making it one of the top performers among the models evaluated.

On testing dataset 1 (Table 3), EnsembleNPPred achieved an accuracy (ACC) of 0.940, which was higher than that of all other models, including NeuroPred-CLQ, which had the next highest ACC at 0.936. Additionally, our model outperformed the other models in terms of the Matthews correlation coefficient (MCC), which was 0.881. The high MCC suggests that EnsembleNPPred has a strong predictive capability with a good balance between sensitivity (Sn = 0.962) and specificity (Sp = 0.918). Notably, the area under the curve (AUC) was the highest among all methods at 0.990, further indicating the robustness of our model in distinguishing neuropeptides from non-neuropeptides.

When evaluated on testing dataset 2 (Table 4), which includes more recent neuropeptide sequences from the NeuroPep 2.0 database, EnsembleNPPred again achieved top-tier performance. It reached an ACC of 0.929 and an MCC of 0.859, surpassing the performance of NeuroPred-PLM, which achieved an ACC of 0.922 and an MCC of 0.845. The precision and recall of our model were both high, at 0.930 and 0.929, respectively, resulting in an F1 score of 0.929, the highest among all compared methods.

Across both independent datasets, EnsembleNPPred consistently demonstrated a better performance, highlighting its robustness and reliability in neuropeptide prediction. Its high accuracy, MCC, and AUC scores suggest not only strong predictive capability but also a well-balanced performance between precision and recall, making it highly effective across different datasets.

The EnsembleNPPred framework combining interpretable handcrafted features with sequence patterns automatically learned through CNN embedding. This hybrid design helps mitigate potential issues such as overfitting, feature redundancy, and limited interpretability, particularly when classifying heterogeneous peptide families. By integrating domain-specific knowledge and data-driven representations, the final model architecture was selected based on its stability, interpretability, and computational efficiency. While promising, the current approach could benefit from future enhancements, such as adopting advanced deep learning paradigms that better capture long-range dependencies and structural complexity in peptide sequences.

### 3.5. Performance Across Diverse Neuropeptide Families

To further assess generalizability, we evaluated the model on a diverse range of neuropeptide families using testing dataset 3, as detailed in Supplementary Data S1. This additional evaluation served to mitigate dataset-specific bias and further assessed the model’s robustness, particularly for well-defined peptide classes. The results demonstrate that the model performs well across a broad range of neuropeptide families. High accuracy was observed for families such as AKH/HRTH/RPCH (99.62%), FMRFamide-related peptide (99.65%), insulin (98.26%), and NPY (99.18%). The overall average accuracy across all families was 91.92%, although performance varied depending on the specific family. Challenges observed in structurally complex, or neuropeptide-like families highlight potential areas for future model improvement through structural feature integration. Certain families, such as chemokine (44%), serpin (37.61%), and nucleobindins (66.67%), exhibited reduced accuracy. These discrepancies may be attributed to factors such as complex post-translational modifications, the presence of multiple isoforms, and dynamic structural conformation [62,63,64,65,66,67,68,69], which complicate classification. It is also worth noting that some families such as chemokines, chromogranin/secretogranin, nucleobindin, serpins, and tenascins are often categorized as “neuropeptide-like” rather than canonical neuropeptides. According to Burbach [1,4], neuropeptide-like peptides may influence neural or physiological activity in similar ways to classical neuropeptides but fail to meet all defining criteria, such as originating from neurons, being secreted in a stimulus-dependent manner, or acting via well-characterized neuropeptide receptors. These deviations are likely to contribute to the reduced classification performance observed in these families.

For benchmarking purposes, we compared our model against the NeuroPred-FRL web server [20], which provides a widely accessible and user-friendly interface. A detailed comparison of predictions generated by NeuroPred-FRL across all neuropeptide families is presented in Supplementary Data S2 and summarized in Supplementary Data S1. On the same evaluation dataset, our proposed model achieved an overall accuracy of 91.92%, while NeuroPred-FRL achieved 71.97%. These results suggest that EnsembleNPPred offers competitive performance under the conditions tested.

Overall, the proposed ensemble approach, which integrates both machine learning and deep learning techniques, demonstrates robust performance across most neuropeptide families and appears particularly effective for classifying well-characterized peptides. The average accuracy of 91.92% across all families suggests that the model is generally useful for neuropeptide prediction. However, the reduced performance observed for structurally complex or atypical families highlights key limitations. Addressing these challenges in future work may require incorporating more advanced structural feature representations and expanding the training dataset to include greater sequence diversity, thereby enhancing generalizability and predictive accuracy.

### 3.6. Evaluation of False Positive Rates for EnsembleNPPred

To assess the false positive rate of EnsembleNPPred, we used antimicrobial peptides from the DRAMP Database [70] as a negative test set (see Supplementary Data S3 for details). EnsembleNPPred demonstrates consistently low false positive rates across multiple AMP categories. Specifically, the model achieved false positive rates of 6.68% for antibacterial, 12.13% for antifungal, and 11.86% for antiviral peptides. These results suggest that EnsembleNPPred provides relatively high specificity and robustness in distinguishing neuropeptides from non-neuropeptide sequences.

To further investigate the nature of the misclassified sequences, a subset of AMP sequences that were predicted as neuropeptides was analyzed for conserved domains using the InterPro domain database (Supplementary Data S3, column L). Interestingly, the majority of these peptides were found to contain domains associated with neuropeptide precursors or neuroendocrine signaling in addition to their known antimicrobial functions. In accordance with the report by Wei et al., neuropeptides and AMPs share common characteristics, i.e., signal sequence, mostly at the N-terminus, and size [12]. Notable examples include the Chromogranin A/B/C domain, neuropeptide-like protein domains, and the Tachykinin domain [71], all of which are associated with classical neuropeptide signaling pathways. Moreover, several other domains identified in the misclassified sequences—e.g., Acyl-CoA-binding protein [72], Chemokine beta/gamma/delta [73], and Pancreatic hormone-like domain [74]—are not traditionally classified as neuropeptide-related but have been reported to be involved in the regulation of the immune system [75]. While the presence of these domains does not confirm neuropeptide function, it raises the possibility that some misclassified AMP sequences may exhibit a domain structure common to that of neuropeptides, which may possibly lead to dual-functionality, as previously reported [11,12].

Taken together, these observations suggest the possibility that certain peptides may exhibit dual-functionality—potentially contributing to both host defense and neuroimmune modulation, particularly in relation with bidirectional gut–brain communication to regulate microbiome population in the gut and immune response mechanisms of the host. However, experimental validation will be necessary to determine the biological relevance of these predictions and to confirm whether these peptides indeed possess neuropeptide-like functions.

## 4. Conclusions

This study presents EnsembleNPPred, a hybrid neuropeptide prediction framework that integrates traditional machine learning (ML) techniques with a deep learning (DL) component using an ensemble voting strategy. By integrating interpretable handcrafted features with CNN-derived embeddings from k-mer-based Word2Vec encoding, the model leverages both domain knowledge and data-driven representations to improve predictive performance and interpretability. The model was evaluated on two independent test sets and demonstrated consistent and competitive results. On the first test set, EnsembleNPPred achieved an accuracy of 0.940 and a Matthews correlation coefficient (MCC) of 0.881. On the second test set, it attained an accuracy of 0.929, an MCC of 0.859, and an F1 score of 0.929, surpassing several existing methods under the tested conditions.

When applied across a wide range of neuropeptide families, EnsembleNPPred maintained strong performance, particularly for well-characterized families such as AKH/HRTH/RPCH, FMRFamide-related peptides, insulin, and NPY. However, reduced accuracy was observed for families such as chemokines, leptin, and serpins. This may be attributed to their complex structural features, including diverse receptor interactions, conformational flexibility, and the presence of multiple isoforms. To address these challenges, future enhancement may focus on incorporating more advanced structural representations and expanding training datasets to better capture conformational diversity and post-translational modifications. One possible direction includes integrating three-dimensional structural data (e.g., predicted tertiary conformations or receptor-binding motifs) potentially using pretrained transformer-based models such as ESM or ProtBERT for representing tertiary conformations or receptor-binding properties. EnsembleNPPred demonstrates strong performance in neuropeptide classification, particularly in canonical families; however, further refinement and broader validation on novel or experimentally derived datasets are still needed. Overall, EnsembleNPPred provides a flexible and interpretable framework for large-scale peptide annotation, with the potential to support research in functional genomics, neurobiology, and therapeutic peptide discovery.

## Figures and Tables

**Table 1:** Percent composition of amino acid based on physicochemical properties.

Physicochemical Property	Positive Data (NPs)	Negative Data (Non-NPs)
Average Length (amino acid residue)	23.93	26.93
% Charged Residues (DEKHR)	24.41	22.81
% Aliphatic Residues (ILV)	16.04	21.78
% Aromatic Residues (FHWY)	12.84	11.22
% Polar Residues (DERKQN)	30.79	28.25
% Neutral Residues (AGHPSTY)	41.27	35.01
% Hydrophobic Residues (CVLIMFW)	27.94	36.74
% Positively Charged Residues (HKR)	12.98	14.82
% Negatively Charged Residues (DE)	11.43	7.99
% Tiny Residues (ACDGST)	36.58	32.01
% Small Residues (EHILKMNPQV)	45.85	53.04
% Large Residues (FRWY)	17.57	14.95

**Table 2:** Classification performance on the training dataset using 10-fold cross-validation.

Model	ACC	MCC	Sn	Sp	AUC	95% ROC CI
SVM	93.819	0.876	0.940	0.936	0.986	[0.973–0.995]
KNN	90.570	0.814	0.870	0.942	0.968	[0.954–0.981]
DT	88.569	0.772	0.876	0.895	0.877	[0.853–0.899]
RF	93.324	0.866	0.930	0.936	0.985	[0.971–0.994]
ET	93.779	0.876	0.938	0.937	0.986	[0.973–0.996]
XGB	91.660	0.833	0.915	0.918	0.972	[0.963–0.984]
DL	93.918	0.878	0.939	0.938	0.986	[0.973–0.996]
Ensemble	93.978	0.880	0.939	0.941	0.987	[0.975–0.996]

**Table 3:** Comparison of performance on independent testing dataset 1.

Method	ACC	MCC	Sn	Sp	AUC
NeuroPpred-Fuse	0.906	0.813	0.882	0.930	0.958
PredNeuroP	0.897	0.794	0.886	0.907	0.954
NeuroPred-FRL	0.900	0.803	0.946	0.854	0.965
NeuroPIpred	0.536	0.074	0.331	0.736	0.581
NeuroPred-CLQ	0.936	0.875	0.897	0.975	0.988
EnsembleNPPred	0.940	0.881	0.962	0.918	0.990

**Table 4:** Comparison of performance on independent testing dataset 2.

Method	ACC	MCC	Precision	Recall	F1
PredNeuroP	0.864	0.738	0.935	0.782	0.852
NeuroPred-FRL	0.861	0.740	0.960	0.757	0.847
NeuroPpred-Fuse	0.905	0.813	0.906	0.908	0.907
NeuroPred-PLM	0.922	0.845	0.907	0.941	0.924
EnsembleNPPred	0.929	0.859	0.930	0.929	0.929

## Data Availability

The data presented in this study are available in the supplementary materials of this article.

## Supplementary Materials

The following supporting data are available online at https://www.mdpi.com/article/10.3390/life15071010/s1, Supplementary Data S1: Assessment of model accuracy across neuropeptide families, Supplementary Data S2: Detailed comparison of predictions generated by NeuroPred-FRL across all neuropeptide families, Supplementary Data S3: Evaluation of false positive rates for EnsembleNPPred across multiple AMP categories.
